# Medicolegal Evaluation of Long-Term Respiratory Functions in Patients Injured Due to Traffic Accidents

**DOI:** 10.7759/cureus.15642

**Published:** 2021-06-14

**Authors:** Esra Dugral, Aydin Sanli, İsmail Ozgur Can

**Affiliations:** 1 Department of Pulmonology, Dokuz Eylul University, Faculty of Medicine, İzmir, TUR; 2 Department of Thorax Surgery, Dokuz Eylul University, Faculty of Medicine, İzmir, TUR; 3 Department of Forensic Medicine, Dokuz Eylul University, Faculty of Medicine, İzmir, TUR

**Keywords:** medicolegal, pulmonary function, traffic accident, chest trauma, forensic medicine

## Abstract

Aim

Blunt chest trauma is a frequent injury in developing countries, with motor vehicle accidents being the most common cause. Most studies about the effects of post-traumatic injuries on pulmonary functions are related to the acute phase. The aim of this study is to compare the effect of injury type on pulmonary function tests as a long-term disability in patients with severe chest trauma due to traffic accidents.

Methods

In our study, 53 patients were admitted to the Forensic Expert Council with the aim of determining the disability ratio at least six months after the traffic accident. All patients who had a respiratory function test because of respiratory symptoms and whose reporting period was completed were appreciated. A retrospective examination of the forensic committee reports, types of injuries, and current pulmonary function test results were analyzed and the data were evaluated by using the Statistical Package for the Social Sciences (SPSS) 22.0 program (IBM Corp, Armonk, NY).

Results

Thirty-two (32) of the patients were male while 21 were female. Their average age was 39.88 ± 15.29. Sixty-six percent (66%; n: 35) of the cases were injured due to in-vehicle traffic accidents, 18.9% (n: 10) due to motorcycle accidents, 15.1% (n: 8) due to non-vehicle traffic accidents. The number of cases with costa fractures was 47 and 74.4% of these cases had three or more rib fractures. The mean forced expiratory volume in 1 second/forced vital capacity ratio (FEV1/FVC-Tiffeneau-Pinelli index) was calculated as 85.3% ± 9.45, and the average FVC was 84.3 ± 14.98%. The average number of rib fractures in all patients was 3.41 ± 2.24. It was observed that tube thoracostomy was performed in seven of 12 cases with FEV1/FVC below 80%, and the average number of rib fractures was 3.75. In 20 cases where the FVC average was below 80%, the mean number of rib fractures was 3.8, and tube thoracostomy was performed in 10 of these cases. The highest FEV1 value was 116%, and the lowest FEV1 value was 35%. The FEV1 value of 23 cases was between 75% and 95%. The highest FEV1/FVC value was 113% and the lowest FEV1/FVC value was 50%. The FEV1/FVC values of 38 cases were between 80% and 100%.

Conclusions

In our study, most patients achieve near-complete recovery in pulmonary function tests; the impact of pre-existing pulmonary compromise on recovery is less known. The number of rib fractures can reflect the severity of the blunt trauma but it would not necessarily predict the resulting pulmonary function. These results are consistent with the previous studies. Further larger prospective studies are required to investigate different factors affecting prognosis.

## Introduction

Injuries that occur in the body as a result of various traumas cannot always heal, may cause sequelae or malfunction, and may affect the life of the person. Disability is defined as the inability to fulfill a life that is considered normal according to gender, age, and cultural and social status due to disability caused by injury or sequela. The World Health Organization (WHO) has defined deficiency in the body as the impairment of psychological, physiological, or anatomical integrity or loss of function or impairment. Disability was defined as the inability of the person to perform the actions accepted within the "normal" limit as a result of the long duration of this deficiency. Most of the disability claims are applications for disability due to injuries due to traffic accidents [1-2].

Trauma is the leading cause of death in the young group under 40 years old. Traffic accidents, falls from a height, and work accidents are the most common causes of blunt trauma. Many organ injuries as a result of trauma can be seen together, but 25% of fatal accidents have thoracic trauma or complications caused by it [3].

Blunt chest trauma is a type of injury that is frequently seen in developed countries and constitutes 10%-15% of all traumas. Motor vehicle accidents are the most common cause of this type of trauma. The risk of serious thoracic injury due to any motor vehicle accident is approximately 7%. In addition, thoracic traumas have been associated with high morbidity and mortality [4-6].

Rib fractures are the most common bone injuries in thoracic trauma and occur in approximately 50% of hospitalized patients after thoracic trauma. The number and location of fractures are important because the higher the number of fractures, the higher the complication rates. In addition, the location of the rib fracture is important in terms of causing injury to the lung, heart, main vessels, mediastinum, pleura, and even bronchi. While hemothorax and pneumothorax are seen as complications in the early period, the possibility of atelectasis increases due to insufficient treatment in the late period.

While people who have had blunt chest trauma are mostly followed with conservative treatments, only 10% of them need surgical intervention. Conservative treatments include pain control, tube thoracostomy, oxygen therapy, and respiratory physiotherapy [7].

Surgery as a treatment option in blunt chest trauma occurs in the presence of certain risk factors that also affect mortality and morbidity. Among them, the number and location of rib fractures, the presence of chronic lung disease or other chronic diseases in the medical history, advanced age, length of stay in intensive care, presence of head trauma, etc. could be counted [8].

In cases with rib fractures, the main diagnostic goal is to detect related complications such as hemopneumothorax, pulmonary contusion, intra-abdominal injury, or major vascular damage [9]. Advances in the treatment and management of blunt chest trauma have reduced the mortality of such injuries in many centers, but the long-term respiratory sequelae of blunt chest trauma continue to be evaluated [10-11]. Although studies are ongoing, most of the important data on blunt chest trauma are related to the acute phase, and there are limited and conflicting information about late sequelae and recovery [12]. In this study, in patients who had severe chest trauma due to a traffic accident and who applied to the department of forensic medicine with a request for disability evaluation, we aimed to examine the long-term effects of injury type on pulmonary function tests and impact on disability degree.

## Materials and methods

In our study, in order to determine the rate of sequelae at least six months after the traffic accident, the report processes of 53 patients who applied to Dokuz Eylül University Research and Practice Hospital Forensic Medicine Department were retrospectively evaluated. Permission was obtained from the local ethics committee for the article. The forensic medical reports were analyzed retrospectively, and the age, accident type and diagnosis, injury types, clinical examinations, and pulmonary function test results were analyzed. The data were processed using the Statistical Package for the Social Sciences (SPSS) 22.0 program (IBM Corp, Armonk, NY) and evaluated statistically.

## Results

Thirty-two (32) of the patients were male while 21 were female. Their average age was 39.88 ± 15.29. Sixty-six percent (66%, n: 35) of the cases were injured due to an in-vehicle traffic accident, 18.9% (n: 10) due to a motorcycle accident, and 15.1% (n: 8) due to a non-vehicle traffic accident. The number of cases with costa fractures was 47, and 74.4% of these cases had three or more rib fractures. The mean forced expiratory volume in 1 second/forced vital capacity ratio (FEV1/FVC-Tiffeneau-Pinelli index) was calculated as 85.3% ± 9.45, and the average FVC was 84.3 ± 14.98%. The average number of rib fractures in all patients was 3.41 ± 2.24. It was observed that tube thoracostomy was performed in seven of 12 cases with FEV1/FVC below 80%, and the average number of rib fractures was 3.75. In 20 cases where the FVC average was below 80%, the mean number of rib fractures was 3.8 and tube thoracostomy was performed in 10 of these cases. The highest FEV1 value was 116% and the lowest FEV1 value was 35%. The FEV1 value of 23 cases was between 75% and 95%. The highest FEV1/FVC value was 113% and the lowest FEV1/FVC value was 50%. The FEV1/FVC values of 38 cases were between 80% and 100%.

**Table 1:** Demographic, characteristics, and main data of the patients FEV1: forced expiratory volume in 1 second; FVC: forced vital capacity

n (number)	53
Age	39.88 ± 15.29
Male	32
Female	21
Accident Type	
In-Vehicle Traffic Accident	35
Motorcycle Accident	10
Non-Vehicle Traffic Accident	8
Costa Fracture Seen	
Number Of Patients	47
Average Rib Fracture	3.41 ± 2.24
FEV_1_/FVC	%85.3 ± 9.45
FVC	%84.3 ± 14,98
FEV_1_/FVC< %80	12
Tube Thoracostomy	7
Average Rib Fracture	3,75
FVC	20
Tube Thoracostomy	10
Average Rib Fracture	3,8

## Discussion

Traumas are among the top causes of death in the young adult group. Chest trauma has been detected in 25% of the people who are exposed to trauma, mostly due to in-vehicle or non-vehicle traffic accidents, and this situation negatively affects mortality and morbidity [13].

Ninety percent (90%) of people with blunt chest trauma are followed up with conservative treatments without resorting to surgical treatments. However, the tube thoracostomy procedure, which can be considered minor surgery, is applied to approximately half of the patients in severe traumas [7]. In our study, tube thoracostomy was performed in 10 of 20 patients with FVC <80% and in seven of 12 patients with FEV1/FVC <80% after severe trauma.

Death may occur within hours following thoracic trauma due to airway obstructions, tracheobronchial injury, ventilation-perfusion disorders due to pneumothorax and hemothorax, cardiac tamponade, and severe bleeding as a result of injury to the main vascular structures [14]. However, 90% of the other hospitalized patients can be discharged with conservative treatments.

Rib fractures are the most common hospitalized group after thoracic trauma. In our study, 47 of 53 patients who had blunt thoracic trauma had rib fractures. The location and number of rib fractures are important because it parallels the increase in complications caused by fractures. As the number of fractures increases, the rate of complications increases. Serious fractures that may occur in the ribs may cause an increase in mortality, as they will cause injury to the lung, pleura, mediastinum, heart, and main vascular structures. In our study, our patients did not have vital organ injuries but had rib fractures, and our average number of rib fractures was found to be 3.41 ± 2.24.

Pain, which increases with coughing or deep breathing, is the most important symptom of rib fractures. Pain must be taken under control because, in the presence of pain, the patient cannot cough enough and cannot breathe deeply, while secretion accumulation and mucus plugs develop, the picture may progress to atelectasis.

Toward the end of the 20th century, the approach to thoracic traumas accelerated in a very good direction. With advances in imaging techniques, the location of the trauma and the damage it causes were determined earlier and accurately while the advancement in surgical methods increased the survival chance of the patients. Clinical improvements in patient care methods have paved the way for recovery without sequelae in people exposed to thoracic trauma with better respiratory support and protection from infections [15].

A spirometer is the most commonly used test to evaluate the ventilation capacity in patients with thoracic trauma. Many institutions, such as the American Thoracic Society (ATS), have published guidelines evaluating the severity of impairment in respiratory functions. In ATS guidelines, it was stated that the workforce was “Full” when FVC (% expected): ≥80, and “Mild (Near Full)” when FVC (% expected) is 60-79. In our study, the FVC was 84.3% ± 14.98, and it was seen that people with thoracic trauma recovered either "Full" or "Mild (Near Full)" according to the results of pulmonary function tests after six months. Likewise, it is accepted by ATS that the workforce is "Full" when FEV1/FVC% is 75 and "Mild (Near Full)" when FEV1/FVC% is 60-74. In our study, FEV1/FVC was found to be 85.3% ± 9.45, and it is seen that they are in the group recovered without sequelae and without loss of workforce [16].

Studies have shown that there is a rapid improvement in all parameters of pulmonary function tests a few months after severe thoracic trauma, and it almost returns to normal after six months [11]. In another study conducted by Amital et al., it was shown that respiratory function tests of the patients were almost completely improved after severe thoracic trauma [12].

In some other studies, it was found that functional residual capacity (FRC) and partial pressure of oxygen (PaO2) decreased when the supine position was switched to patients who developed pulmonary contusion after thoracic trauma even after a few years. It has been shown that lung functions recover within six months in patients without pulmonary contusion even with permanent chest wall deformity [17].

Similarly, in our study, it was found that there was an almost complete improvement in respiratory function tests performed at least six months after thoracic trauma.

Toward the end of the 20th century, the chance of survival after trauma and even recovery without sequelae increased with the advances in diagnosis and treatment in the medical world.

A limitation is that our study could not be fully performed because of a retrospective clinical data screening and because the medical history of the patients was not recorded in the hospital records. We analyzed reports from 53 patients of the adult cohort. Statistically speaking, this sample appears small. Thus, the sample size is not a limiting factor although in clinical and medicolegal research, the more samples the better. In addition, similar published studies have similar sample sizes if not smaller.

## Conclusions

In our study, we concluded that long-term respiratory failure is very rare after severe chest trauma, and patients recovered rapidly and almost completely according to pulmonary function tests at the end of six months. Our other conclusion is that the number of rib fractures can give information about the severity of blunt chest trauma but is insufficient in predicting post-traumatic long-term pulmonary function. These results showed how important early diagnosis and rapid/accurate medical interventions are in thoracic trauma and disability rate. Although our results are compatible with the literature, larger-scale prospective studies are needed to investigate different factors affecting prognosis and medicolegal evaluations in the long term.
